# ADHD, Subtypes and Cognitive Performance: WISC-V as a Support Tool for Diagnostic Assessment

**DOI:** 10.62641/aep.v54i3.2122

**Published:** 2026-06-15

**Authors:** Rocío Lavigne-Cerván, Joshua Collado-Valero, Ignasi Navarro-Soria, Megan Rosales-Gómez, Manuel Torrecillas-Martínez

**Affiliations:** ^1^Department of Developmental and Educational Psychology, University of Málaga, 29004 Málaga, Spain; ^2^Department of Developmental Psychology and Didactics, Faculty of Education, University of Alicante, 03690 Alicante, Spain

**Keywords:** ADHD, cognitive profile, processing speed, WISC, working memory

## Abstract

**Background:**

Attention-Deficit/Hyperactivity Disorder (ADHD) is a neurodevelopmental condition characterized by impairments in working memory (WM) and processing speed (PS). Previous studies using earlier editions of the Wechsler Intelligence Scale for Children (WISC) have identified a cognitive response pattern defined by lower Cognitive Proficiency Index (CPI). However, evidence using the Wechsler Intelligence Scale for Children-Fifth Edition (WISC-V) remains limited. This study aimed to examine whether the WISC-V identifies a characteristic cognitive profile in ADHD and to evaluate its discriminative capacity.

**Methods:**

The sample consisted of 188 participants aged 6–16 years, including a control group (n = 56) and two clinical ADHD groups: The combined presentation of ADHD (ADHD-C; n = 57) and the predominantly inattentive presentation of ADHD (ADHD-I; n = 75). Cognitive functioning was assessed using the WISC-V. Group differences were examined using Multivariate and Univariate Analyses of Covariance (MANCOVA/ANCOVA). Binary logistic regression analyses were conducted to evaluate the predictive capacity of WISC-V.

**Results:**

Compared with controls, the clinical ADHD group showed significant multivariate differences. Univariate analyses revealed significantly lower performance in the working memory index (WMI; *p* < 0.001), processing speed index (PSI; *p* < 0.001), CPI (*p* < 0.001), and Full-Scale Intelligence Quotient (FSIQ; *p* = 0.005), in the clinical ADHD group. Differences between ADHD-C and ADHD-I were observed, with the ADHD-I group showing lower scores in WMI (*p* = 0.016), PSI (*p* < 0.001), CPI (*p* < 0.001), and FSIQ (*p* = 0.016). Logistic regression analyses indicated that WMI (B = −0.067; *p* < 0.001), PSI (B = −0.043; *p* = 0.007), and CPI (B = −0.091; *p* < 0.001) significantly predicted control versus clinical ADHD group membership. When comparing ADHD presentations, PSI (B = −0.055; *p* = 0.001) and CPI (B = −0.063; *p* < 0.001) emerged as significant predictors.

**Conclusions:**

The findings support the use of the WISC-V as a complementary tool in the diagnostic assessment of ADHD.

## Introduction

With the consolidation of psychometrics and the growing interest in specific patterns of cognitive functioning, the first attempts emerged to identify concrete cognitive traits through standardized instruments [1, 2]. These early efforts laid the groundwork for more detailed and systematic research into how cognitive abilities are expressed in children and adolescents. Subsequently, Bannatyne [3] conducted the first analysis of cognitive profiles using the Wechsler Intelligence Scale for Children (WISC), which is considered one of the most widely used psychometric tests worldwide [4], thereby providing a structured framework for studying individual differences in cognitive functioning. As research progressed, and with the development of new models of cognitive processing applied to neurodevelopmental disorders, such as Autism Spectrum Disorder (ASD) [5] and Language Disorder (LD) [6], scholars such as Prifitera and Dersh [7] highlighted the need to establish a specific cognitive profile for Attention-Deficit/Hyperactivity Disorder (ADHD).

ADHD is a neurodevelopmental disorder characterized by a persistent pattern of inattention, hyperactivity, and impulsivity that significantly interferes with an individual’s personal, academic, social, or occupational functioning [8]. However, these core symptoms represent only the observable manifestation of a more complex underlying clinical condition, in which multiple neurobiological processes are involved [9]. Over the past decades, alternative neuropsychological approaches have shifted the focus toward higher-order cognitive processes, particularly executive functions (EF) [10, 11]. These include behavioral inhibition, working memory (WM), emotional self-regulation, cognitive flexibility, planning, and organization [11, 12]. This perspective has contributed to the understanding that ADHD involves not only difficulties in attention, hyperactivity, and impulsivity, but also a specific pattern of executive functioning reflected in distinct cognitive profiles.

Since then, many professionals have considered it appropriate to implement the WISC to assess the cognitive functioning of individuals with ADHD [13, 14]. Over time, the repeated administration of this scale to individuals with ADHD has led to the hypothesis of an existing and characteristic response pattern [15]. Thus, the third edition of the WISC [16] made it possible to identify distinctive cognitive performance within this population. Studies have shown significant impairments in the Freedom from Distractibility Index (FDI), which assesses sustained attention and WM abilities; as well as in indices related to visuospatial skills and processing speed (PS) [17, 18]. The characteristic response pattern is reflected in the Symbol Search, Coding, Arithmetic, Digit Span (SCAD) and Arithmetic, Coding, Information, Digit Span (ACID) profiles, which are used as indicators of deficits in EF and attentional control. Taken together, these findings strengthen the evidence that ADHD is associated with specific alterations in cognitive processes, particularly behavioral inhibition, WM, and PS [19, 20].

The modifications introduced in the fourth edition of the WISC [21] substantially increased its sensitivity for detecting cognitive characteristics of ADHD [22], while also providing evidence of adequate structural validity in ADHD populations [23]. Findings consistently show lower performance on the working memory index (WMI), which reflects the ability to actively manipulate information, and on the processing speed index (PSI), associated with the speed and automatization of cognitive processes [24, 25, 26]. In contrast, scores on the Verbal Comprehension Index (VCI) and the Perceptual Reasoning Index (PRI), indicators of conceptual reasoning and efficiency in visuospatial processing, generally remain within typical ranges [21, 27, 28, 29].

Therefore, the results of the scale assessment were characterized by higher scores in VCI and PRI, and lower scores in WMI and PSI (VCI ≈ PRI > WMI ≈ PSI). Some authors have described this pattern more visually as a “cognitive step”, since the declines in WMI and PSI are especially pronounced when represented graphically [30, 31, 32]. These findings are consistent with those reported in the recent systematic review conducted by Lavigne-Cerván *et al*. [15], which analyzed twenty-seven ADHD studies using the WISC over a period of approximately thirty years.

The discrepancy among these index scores becomes even more evident when the primary indices are integrated into composite indices. The General Ability Index (GAI) provides an estimate of general reasoning ability based on verbal and nonverbal tasks, excluding WM and PS, whereas the Cognitive Proficiency Index (CPI) consolidates performance in these latter domains into a single score. Consistently, the results showed higher GAI scores and lower CPI scores (GAI > CPI) [27, 29].

Although isolated WISC results should not be considered a diagnostic tool for neurodevelopmental disorders such as ADHD [33], they can nevertheless provide valuable complementary information [15]. There is a certain degree of consensus within the scientific community that the cognitive profile associated with ADHD may assist the diagnostic process [34] and serve as a prognostic and predictive tool [35]. The Wechsler Intelligence Scale for Children-Fifth Edition (WISC-V) has demonstrated high reliability coefficients (α = 0.84–0.95; test-retest = 0.79–0.90) and a solid factorial structure supporting its construct validity and diagnostic utility [36]. Likewise, it shows adequate convergent and discriminant validity, particularly as a supporting instrument in the assessment of ADHD and other neurodevelopmental difficulties [37, 38, 39].

The WISC-V includes the VCI, WMI, and the PSI; however, unlike the previous version, the PRI was divided into two new indices: Visual Spatial Index (VSI) and Fluid Reasoning Index (FRI) [36]. Despite the changes, shortly after its publication, individual studies began to emerge that successfully demonstrated the presence of an ADHD- related cognitive profile using the WISC-V [14, 40]. Although the amount of empirical evidence on the ADHD cognitive profile derived from the WISC-V remains considerably more limited than that available for its two previous versions, a response pattern characterized by marked deficits in WM and PS can also be observed (VCI ≈ VSI ≈ FRI > WMI ≈ PSI). This pattern suggests that the “cognitive step” is preserved despite the restructuring of the subscales [41].

Unfortunately, a decade after the publication of the WISC-V, the amount of empirical evidence available to date on cognitive profiles remains limited. The fact that the number of published studies on this topic is still insufficient to support the development of a systematic review highlights the need to replicate and expand research in order to consolidate, broaden, and deepen scientific understanding. Therefore, the main objective of the present study is to examine whether the WISC-V makes it possible to identify a characteristic cognitive profile of ADHD. Additionally, the study aims to incorporate still underexplored aspects, to assess its discriminative capacity across ADHD subtypes, and to analyze the predictive capacity of its results.

## Materials and Methods

### Participants

The total sample consisted of 188 participants aged between 6 and 16 years (Mean (M) = 10.04 years, Standard Deviation (SD) = 2.91 years). The control group included 56 participants in the same age range (M = 10.82 years, SD = 2.99 years). The clinical ADHD group consisted of 132 participants diagnosed with ADHD by a qualified specialist, with a similar age range (M = 9.71 years, SD = 2.82 years). Both the control and clinical ADHD groups were recruited from a private neuropediatric and psychoeducational clinic in Málaga (Spain).

For the control group, eligibility for inclusion required participants to be between 6 and 16 years. The exclusion criteria were defined as follows: a previous diagnosis of ADHD; meeting the diagnostic criteria for ADHD [8]; presence of symptoms of Intellectual Developmental Disorder (IDD; Intelligence Quotient (IQ) < 70) [8, 42]; presence of symptoms of ASD or LD [8, 42]; and presence of symptoms of severe mental disorders (e.g., psychosis) or severe medical conditions (e.g., epilepsy).

For the clinical ADHD group, eligibility for inclusion required participants to be between 6 and 16 years and to have a diagnosis of ADHD established by a qualified professional. The exclusion criteria were defined as follows: presence of symptoms of IDD (IQ < 70) [8, 42]; presence of symptoms of ASD or LD [8, 42]; and presence of symptoms of severe mental disorders (e.g., psychosis) or severe medical conditions (e.g., epilepsy).

### Instruments

Clinical assessment and the initial diagnostic process were conducted prior to the present study by a qualified professional at a private neuropediatric and psychoeducational clinic in Málaga (Spain). For the purpose of the present research, and in collaboration with the aforementioned clinic, a semi-structured interview was conducted by specialized clinicians from the research group. This interview involved a review of each participant’s clinical history and interviews with the child, their relatives, and teachers to verify compliance with the diagnostic criteria established by The Diagnostic and Statistical Manual of Mental Disorders, Fifth Edition, Text Revision (DSM-5-TR) [8].

The cognitive assessment in the present study was carried out by specialized clinicians from the research group using the WISC-V [36], a psychometric test designed to assess the cognitive functioning of children and adolescents aged 6 to 16 years and 11 months. It comprises ten core subtests (M = 10, SD = 3) that form five primary indices (M = 100, SD = 15): VCI, VSI, FRI, WMI, and PSI. In addition, it provides two composite indices: GAI and CPI with the same metric (M = 100, SD = 15), and a Full-Scale Intelligence Quotient (FSIQ).

### Procedure

A non-probability convenience sampling strategy was employed due to the need to apply specific inclusion criteria. As previously indicated, both the control and clinical groups were recruited from a private neuropediatric and psychoeducational clinic in Málaga (Spain). With the appropriate authorization from the aforementioned clinic, the research team accessed an approximate total of 502 clinical reports conducted between September 2023 and December 2024. Following the application of the established inclusion and exclusion criteria, 276 cases were excluded due to the presence of comorbidities that could compromise the integrity of the study, and an additional 38 cases were excluded for not meeting the required age range. Therefore, the final sample included in the present study consisted of a total of 188 participants.

In collaboration with the aforementioned clinic, diagnostic reports and clinical histories were reviewed, and each participant, together with their family members and teachers, was interviewed, by specialized clinicians from the research group, to verify compliance with DSM-5-TR diagnostic criteria [8]. Based on this information, participants were assigned to the control or clinical ADHD groups, with two clinical ADHD subgroups: the combined presentation of ADHD (ADHD-C; n = 57) and the predominantly inattentive presentation of ADHD (ADHD-I; n = 75). No cases met criteria for the hyperactive/impulsive presentation of ADHD (ADHD-HI), which is not uncommon in routine clinical samples. Accordingly, no ADHD-HI subgroup was formed. This distribution is consistent with the findings reported in the systematic review by Lavigne-Cerván *et al*. [15], in which most WISC-based ADHD studies either did not include a separate ADHD-HI subgroup or reported very few cases with that presentation. Following group assignment, the WISC-V was administered. Data collection was conducted between September 2023 and December 2024.

Family members and teachers were notified through the informed consent procedure that participation in the study was voluntary and confidential, and all agreed to take part. The study was approved by the Ethics Committee of The University of Alicante (UA-2023-06-30_1) and conducted in accordance with the principles of the Declaration of Helsinki.

### Data Analysis

The distribution of the sample was examined, and the Kolmogorov-Smirnov test confirmed normality, allowing the use of parametric analyses. Descriptive statistics (frequencies and percentages) were calculated for sex and age. Chi-square tests were used to examine sex differences across the control, ADHD-C, and ADHD-I groups. To analyze the differences between these two clinical ADHD groups and age, an analysis of variance (ANOVA) was applied. Additional descriptive statistics (M and SD) were calculated for WISC-V scores, including the primary indices (VCI, VSI, FRI, WMI, and PSI), the composite indices (GAI and CPI), and FSIQ.

A Multivariate Analyses of Covariance (MANCOVA) was performed for both the primary indices and the composite indices. In the first set of analyses, the independent variable was group membership (control vs. clinical ADHD). In a second set, the same analyses were conducted comparing the two clinical ADHD subgroups (ADHD-C vs. ADHD-I). Sex and age were included as covariates. In addition, an Univariate Analyses of Covariance (ANCOVA) was conducted for each group comparison using FSIQ as the dependent variable. Effect sizes were calculated using partial eta squared (η^2^p), with interpretation following Cohen’s criteria [43].

Finally, four binary logistic regression analyses with a forward stepwise (Wald) method were conducted. The first two models compared the clinical ADHD and control groups: one using the WISC-V primary index scores as predictor variables, and another using the composite indices. The remaining two models compared ADHD presentations, ADHD-C versus ADHD-I, again using the primary index scores and the composite indices as predictors. Statistical significance was set at α = 0.05 (two-tailed). All analyses were performed using the Statistical Package for the Social Sciences (SPSS; Version 25.0 and 27.0; IBM Corp., Armonk, NY, USA).

## Results

### Descriptive Analysis: Sex, Age, and WISC-V Scores (M and SD)

**Fig. 1.**
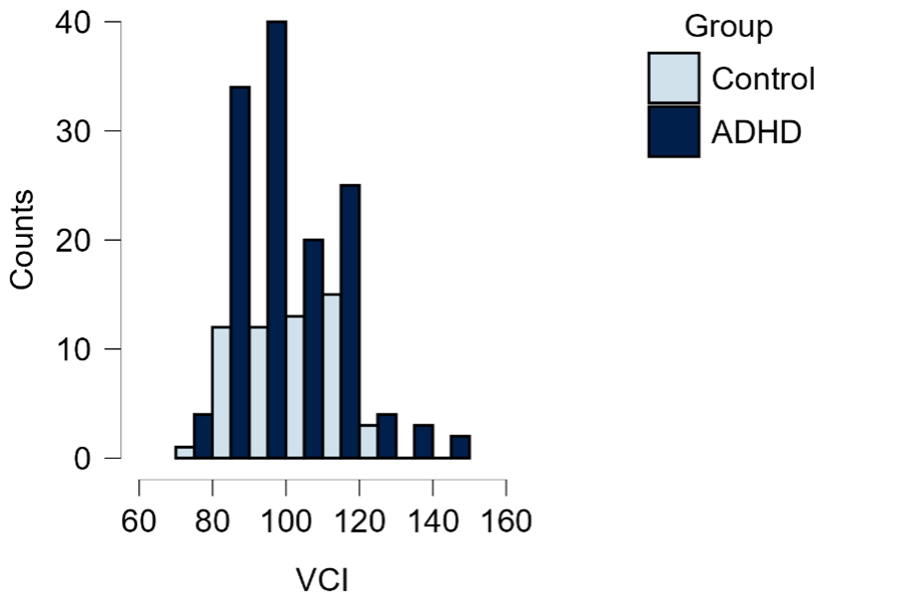
**VCI of the control and clinical ADHD groups**. VCI, Verbal Comprehension Index; ADHD, Attention-Deficit/Hyperactivity Disorder.

**Fig. 2.**
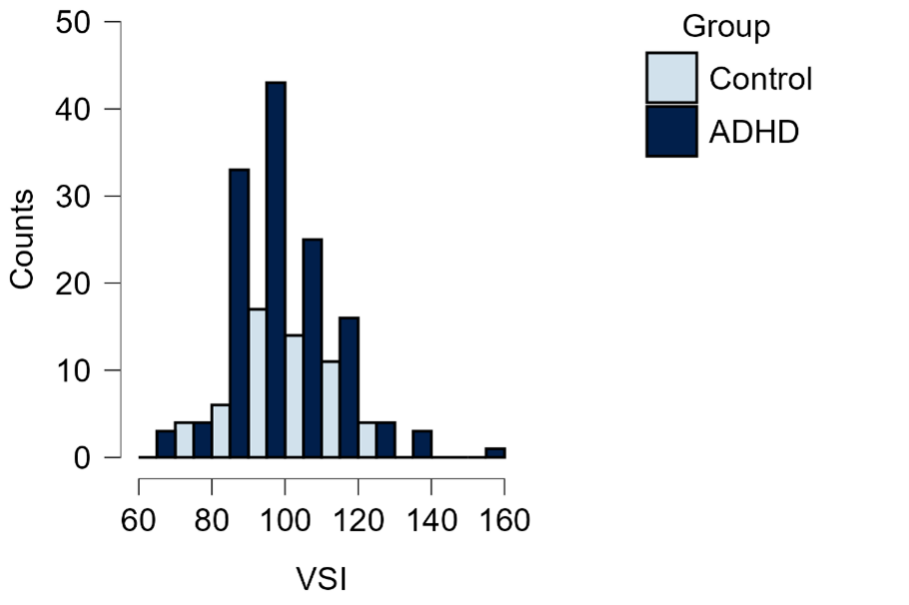
**VSI of the control and clinical ADHD groups**. VSI, Visual Spatial Index; ADHD, Attention-Deficit/Hyperactivity Disorder.

**Fig. 3.**
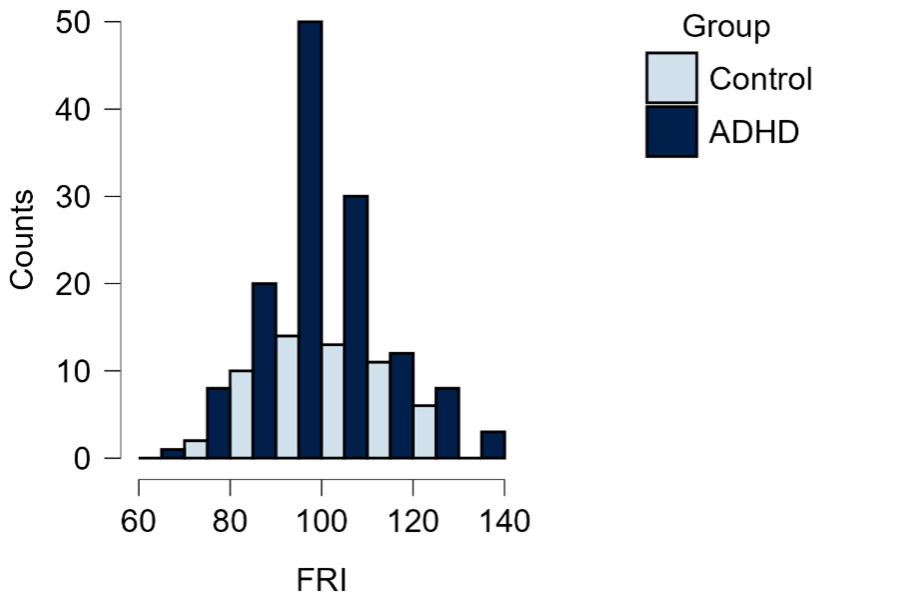
**FRI of the control and clinical ADHD groups**. FRI, Fluid Reasoning Index; ADHD, Attention-Deficit/Hyperactivity Disorder.

**Fig. 4.**
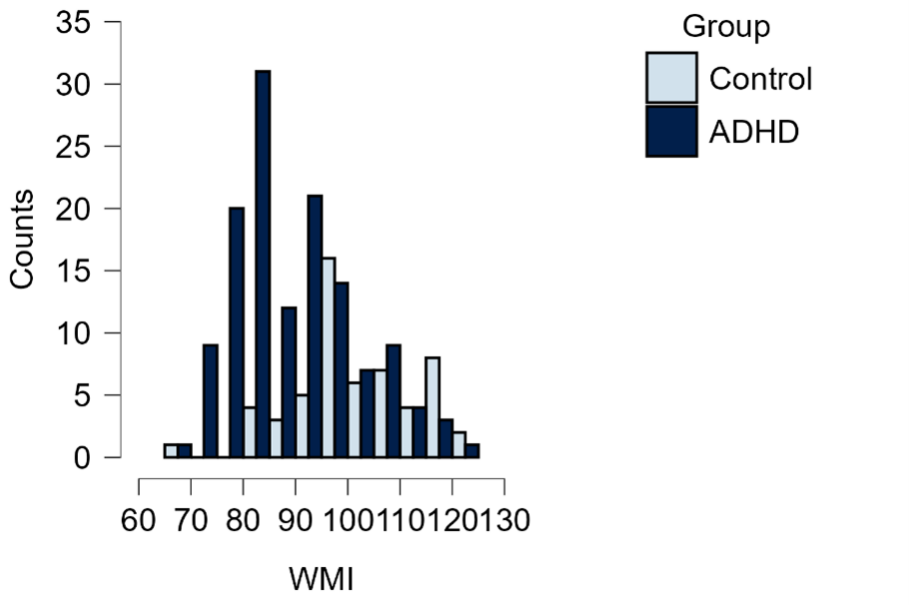
**WMI of the Control and clinical ADHD groups**. WMI, working memory index; ADHD, Attention-Deficit/Hyperactivity Disorder.

**Fig. 5.**
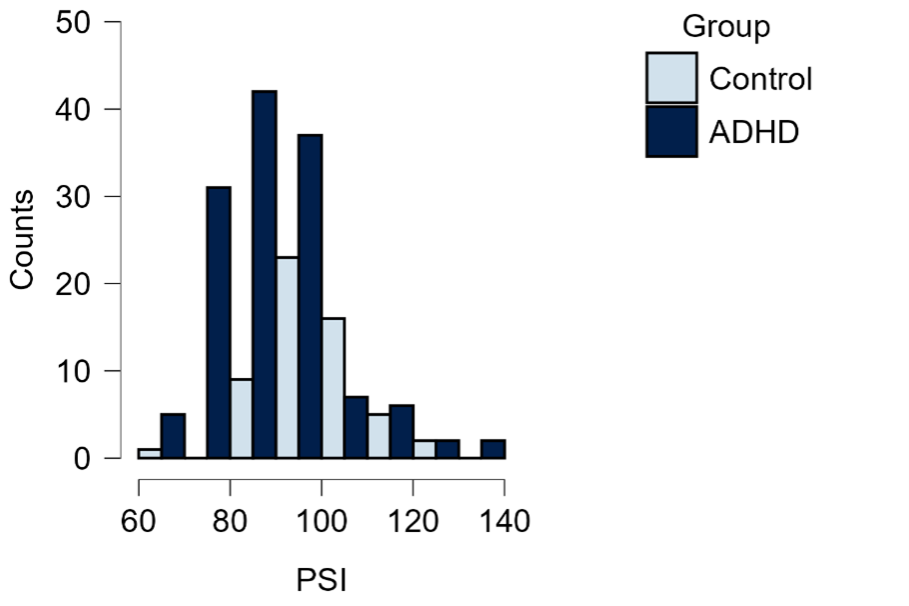
**PSI of the control and clinical ADHD groups**. PSI, processing speed index; ADHD, Attention-Deficit/Hyperactivity Disorder.

**Fig. 6.**
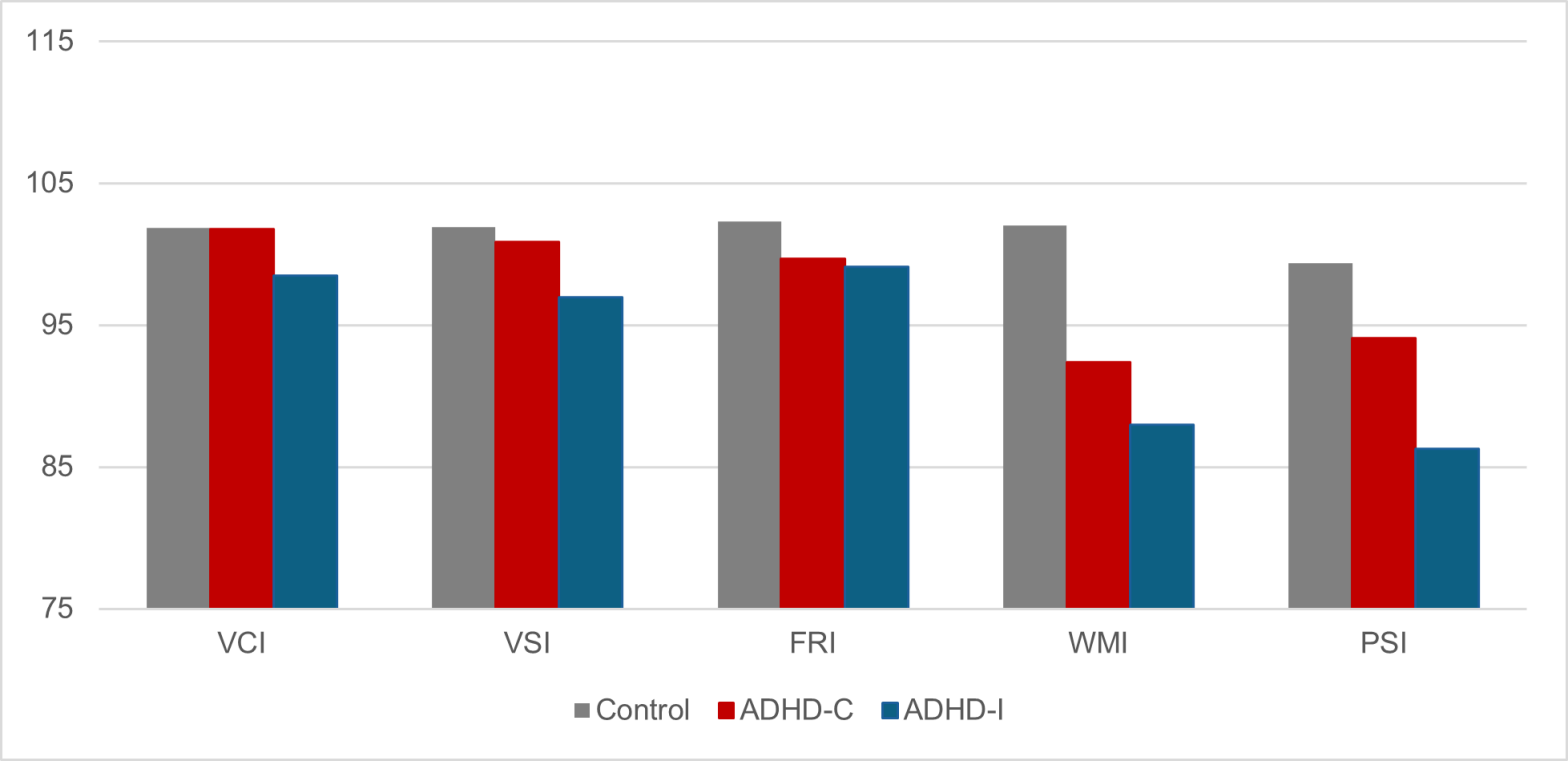
**Cognitive profile on the WISC-V for the control group, ADHD-C, and ADHD-I**. VCI, Verbal Comprehension index; VSI, Visual Spatial Index; FRI, Fluid Reasoning Index; WMI, working memory index; PSI, processing speed index; ADHD, Attention-Deficit/Hyperactivity Disorder; ADHD-C, combined presentation of ADHD; ADHD-I, predominantly inattentive presentation of ADHD; WISC-V, Wechsler Intelligence Scale for Children-Fifth Edition.

The sample consisted of 188 participants aged between 6 and 16 years (M = 10.04, SD = 2.91). Regarding diagnostic distribution, 56 participants were included in the control group, 57 in the ADHD-C group, and 75 in the ADHD-I group. With respect to sex, 145 participants (77.1%) were male and 43 (22.9%) were female. The chi-square test showed no significant differences (*p* = 0.759) in sex distribution between the control and clinical ADHD groups (see Table 1). Age distribution was also examined across groups. The ANOVA test revealed significant differences between the groups and age (*p* = 0.006). In the post hoc test (Tukey’s adjusted), it was found that the groups that differed statistically were the control group and the combined group (*p* = 0.004), while there were no differences between the control group and the inattentive group (*p* = 0.402) or between the inattentive group and the combined group (*p* = 0.085) (see Table 1). Additionally, means and standard deviations were calculated for WISC-V scores across the primary indices, composite indices, and FSIQ (see Tables 1,2). Some of the M and SD of the control group, the clinical ADHD groups, ADHD-C and ADHD-I, are presented graphically for visual comparison (see Figs. 1,2,3,4,5,6).

**Table 1.** **Demographic characteristics by group: sex and age**.

	Control	ADHD-C	ADHD-I	Test value	*p*
Male, n (%)	44 (78.57)	48 (84.21)	53 (70.67)	χ² (2) = 3.462	0.177
Age (M ± SD)	10.82 ± 2.99	9.10 ± 2.41	10.17 ± 3.03	F (2, 185) = 5.275	0.006

ADHD, Attention-Deficit/Hyperactivity Disorder; ADHD-C, combined presentation of ADHD; ADHD-I, predominantly inattentive presentation of ADHD.

**Table 2.** **Comparison of WISC-V index scores between control and clinical groups**.

Indices	Control (n = 56)	Clinical (n = 132)	F (1, 184)	*η*^2^p
	M ± SD	M ± SD		
VCI	102.10 ± 1.87	99.82 ± 1.21	1.03	0.006
VSI	101.71 ± 1.87	98.75 ± 1.21	1.75	0.009
FRI	101.99 ± 1.78	99.46 ± 1.15	1.4	0.008
WMI	101.63 ± 1.60	90.03 ± 1.04	36.37***	0.165
PSI	99.36 ± 1.66	89.64 ± 1.08	23.71***	0.114
GAI	101.92 ± 1.72	99.09 ± 1.11	1.89	0.01
CPI	100.45 ± 1.57	87.64 ± 1.02	46.20***	0.201
FSIQ	100.76 ± 1.65	95.15 ± 1.07	8.07**	0.042

M, Mean; SD, Standard Deviation; VCI, Verbal Comprehension Index; VSI, Visual Spatial Index; FRI, Fluid Reasoning Index; WMI, working memory index; PSI, processing speed index; GAI, General Ability Index; CPI, Cognitive Proficiency Index; FSIQ, Full-Scale Intelligence Quotient; WISC-V, Wechsler Intelligence Scale for Children-Fifth Edition; ** *p* < 0.01; *** *p* < 0.001.

### Differences Between Control and Clinical Groups: MANCOVA and ANCOVA Results

Assumptions of normality, linearity, and homogeneity of variances were tested for all analyses, and no violations were observed (all *p* values > 0.050). A MANCOVA, using Pillai’s Trace, was conducted to examine differences between the control and clinical groups across the primary indices, adjusting for age and sex. The multivariate analysis revealed significant differences between the control and clinical groups (Pillai’s Trace = 0.218, F (5, 180) = 10.04, *p* < 0.001, η^2^p = 0.218). No significant multivariate effects were observed for age or sex.

In the univariate analyses adjusted for age and sex, significant group differences were found for the WMI (F (1, 184) = 36.37, *p* < 0.001, η^2^p = 0.165) and PSI (F (1, 184) = 23.71, *p* < 0.001, η^2^p = 0.114). No significant differences were observed for VCI, VSI, or FRI (see Table 2).

Furthermore, a MANCOVA (Pillai’s Trace) was also conducted to examine whether group differences were present in the composite indices, adjusting for age and sex. The multivariate analysis revealed a significant effect between the control and clinical groups (Pillai’s Trace = 0.218, F (2, 183) = 25.57, *p* < 0.001, η^2^p = 0.218). No significant multivariate effects were observed for age or sex. In the univariate analyses adjusted for age and sex, significant group differences were found for the CPI (F (1, 184) = 46.20, *p* < 0.001, η^2^p = 0.201). No significant differences were observed for the GAI. For the FSIQ, an ANCOVA was conducted controlling for age and sex. Significant differences were observed between the control and clinical groups (F (1, 184) = 8.07, *p* = 0.005, η^2^p = 0.042.)

### Differences Between ADHD-C and ADHD-I: MANCOVA and ANCOVA Results

A MANCOVA (Pillai’s Trace) was also performed to evaluate differences between the ADHD-C and ADHD-I groups across the primary indices, adjusting for age and sex. The multivariate analysis showed significant differences between ADHD-C and ADHD-I (Pillai’s Trace = 0.118, F (5, 124) = 3.33, *p* = 0.007, η^2^p = 0.118). No significant multivariate effects were observed for age or sex. In the univariate analyses adjusted for age and sex, significant differences between groups were found for the WMI (F (1, 128) = 5.92, *p* = 0.016, η^2^p = 0.044) and the PSI (F (1, 128) = 12.86, *p* < 0.001, η^2^p = 0.091). No significant differences were observed for the VCI, VSI, or FRI (see Table 3).

**Table 3.** **Comparison of WISC-V index scores between ADHD-C and ADHD-I groups**.

Indices	ADHD-C (n = 57)	ADHD-I (n = 75)	F (1, 128)	*η*^2^p
	M ± SD	M ± SD		
VCI	101.83 ± 1.92	98.46 ± 1.67	1.7	0.013
VSI	101.16 ± 1.92	96.76 ± 1.67	2.89	0.022
FRI	99.87 ± 1.83	98.93 ± 1.59	0.15	0.001
WMI	92.81 ± 1.58	87.64 ± 1.37	5.92*	0.044
PSI	94.33 ± 1.71	86.10 ± 1.48	12.86***	0.091
GAI	101.47 ± 1.80	97.27 ± 1.56	3.03	0.023
CPI	91.91 ± 1.51	84.25 ± 1.31	14.17***	0.1
FSIQ	98.32 ± 1.70	92.73 ± 1.48	5.94*	0.044

M, Mean; SD, Standard Deviation; VCI, Verbal Comprehension Index; VSI, Visual Spatial Index; FRI, Fluid Reasoning Index; WMI, working memory index; PSI, processing speed index; GAI, General Ability Index; CPI, Cognitive Proficiency Index; FSIQ, Full-Scale Intelligence Quotient; ADHD, Attention-Deficit/Hyperactivity Disorder; ADHD-C, combined presentation of ADHD; ADHD-I, predominantly inattentive presentation of ADHD; WISC-V, Wechsler Intelligence Scale for Children-Fifth Edition; * *p* < 0.05; *** *p* < 0.001.

A MANCOVA, using Pillai’s Trace, was also conducted to examine whether this difference was present in the composite indices, adjusting for age and sex. The multivariate analysis revealed a significant effect between the ADHD-C and ADHD-I groups (Pillai’s Trace = 0.100, F (2, 127) = 7.03, *p* = 0.001, η^2^p = 0.100). No significant multivariate effects were observed for age or sex. In the univariate analyses adjusted for age and sex, significant differences between groups were found for CPI (F (1, 128) = 14.17, *p* < 0.001, η^2^p = 0.100). No significant differences were observed for the GAI. For the FSIQ, an ANCOVA was conducted controlling for age and sex. Significant differences were observed between groups (F (1, 128) = 5.94, *p* = 0.016, η^2^p = 0.044).

### Prediction of Group Membership (Control vs. Clinical): Binary Logistic Regression

The binary logistic regression analyses were used to determine the predictive capacity of the WISC-V primary indices for classifying participants into the control (0) or clinical (1) groups. The model was significant, (χ^2^(2) = 43.208, *p* < 0.001, R^2^ = 0.292) and reliably distinguished between groups, correctly classifying 75.5% of the cases. The model included the WMI (B = -0.067; *p* < 0.001) and PSI (B = -0.043; *p* = 0.007) as predictor variables (see Table 4).

**Table 4.** **Predictors of control vs. clinical group membership**.

Model	Predictor	B	SE	Wald	*p*	OR	95% CI
							LL–UL
Primary index scores	WMI	-0.067	0.015	18.519	<0.001	0.936	0.908–0.964
	PSI	-0.043	0.016	7.278	0.007	0.958	0.929–0.988
	Constant	11.261	1.918	34.49	<0.001	77,768.63	
Composite scores	CPI	-0.091	0.016	31.417	<0.001	0.913	0.885–0.943
	Constant	9.368	1.555	36.293	<0.001	11,706.36	

B, regression coefficient; SE, standard error; OR, odds ratio; CI, confidence interval; LL, lower limit; UL, upper limit;. WMI, working memory index; PSI, processing speed index; CPI, Cognitive Proficiency Index.

A second predictive model was obtained using the composite indices (CPI and GAI). In this analysis, only the CPI contributed significantly to classification, (χ^2^(1) = 42.327, *p* < 0.001, R^2^ = 0.286). This model correctly classified 76.6% of the cases (see Table 4).

### Prediction of ADHD Presentation (ADHD-C vs. ADHD-I): Binary Logistic Regression

A different pattern emerged when classifying participants with ADHD into the combined (0) and inattentive (1) presentations, while also considering demographic variables such as sex and age. The overall model, including the WISC-V primary index scores, sex, and age, was statistically significant (χ^2^ (1) = 21.38, *p* < 0.001, R^2^ = 0.201) and correctly classified 67.4% of participants as either ADHD-C or ADHD-I. Within this model, PSI, sex (men) and age emerged as significant predictors (see Table 5).

**Table 5.** **Predictors of ADHD-C vs. ADHD-I classification**.

Model	Predictor	B	SE	Wald	*p*	OR	95% CI
							LL–UL
Primary index scores	PSI	-0.055	0.017	10.391	<0.001	0.947	0.916–0.979
	Sex (male)	1.057	0.493	4.603	0.032	2.879	1.096–7.563
	Age	0.146	0.07	4.414	0.036	1.158	1.01–1.327
	Constant	3.547	1.644	4.658	0.031	34.715	
Composite scores	CPI	-0.063	0.019	11.583	<0.001	0.939	0.905–0.974
	Sex (male)	1.035	0.489	4.475	0.034	2.815	1.079–7.346
	Age	0.172	0.072	5.669	0.017	1.188	1.031–1.369
	Constant	3.926	1.674	5.504	0.019	50.718	

B, regression coefficient; SE, standard error; OR, odds ratio; CI, confidence interval; LL, lower limit; UL, upper limit; PSI, processing speed index; CPI, Cognitive Proficiency Index; ADHD, Attention-Deficit/Hyperactivity Disorder; ADHD-C, combined presentation of ADHD; ADHD-I, predominantly inattentive presentation of ADHD.

A second binary logistic regression analysis was conducted using the WISC-V composite indices, along with sex and age. This model was also statistically significant, (χ^2^ (1) = 22.53, *p* < 0.001, R^2^ = 0.211) and correctly classified 66.7% of cases. In this analysis, the CPI, sex (male), and age emerged as significant predictors (see Table 5).

## Discussion

The main objective of this study was to examine whether the WISC-V makes it possible to identify a characteristic cognitive profile associated with ADHD and, additionally, to assess its discriminative capacity across presentations. The findings provide consistent evidence in both respects.

First, the comparison between the clinical and control groups revealed clear differences in cognitive indices linked to executive functioning, consistent with previous research [35, 44, 45, 46]. Within this pattern, the WMI showed a large effect size among the primary indices, reinforcing the evidence that associates WM deficits with the core features of ADHD [47, 48]. Converging with this result, significant differences were also found in the PSI with moderate effects, in line with studies that describe reduced PS as a frequent cognitive manifestation of the disorder [49, 50].

Moreover, the CPI composite index showed the strongest group difference of all indices examined, consistent with recent studies highlighting its usefulness as a concise summary of the cognitive deficits typically observed in ADHD [29, 51]. Accordingly, the characteristic cognitive pattern (VCI ≈ VSI ≈ FRI > WMI ≈ PSI) replicates the accumulated evidence reported for the WISC-V [48] as well as for earlier editions of the scale [52, 53] and aligns with the systematic review by Lavigne-Cerván *et al*. [15].

Regarding the comparison between clinical subtypes, significant but smaller differences were observed in the same indices, suggesting that the cognitive alterations are shared by both presentations but expressed with different intensities. Specifically, the ADHD-I group showed greater impairment in PSI, WMI, and CPI, which is consistent with studies that highlight reduced PS, WM, and cognitive efficiency as distinctive features of the inattentive profile [54, 55]. Although these differences are moderate, they provide added clinical value for diagnostic differentiation within the ADHD spectrum.

Finally, when controlling for age and sex, the predictive analyses further reinforce the relevance of the CPI as a cognitive marker of the clinical group, given that lower CPI scores were associated with higher odds of an ADHD diagnosis. Specifically, the WMI and the PSI, which constitute the CPI, also increased the probability of classification.

Furthermore, both the PSI and the CPI yielded useful discriminative performance between presentations, as lower values increased the likelihood of the ADHD-I presentation. Taken together, these findings support the use of the WISC-V as a complementary tool to clinical judgment in contexts involving complex diagnostic decisions [34, 35, 39].

From an applied perspective, identifying a stable cognitive profile, characterized by lower performance in WM and PS, allows for a more precise clinical interpretation of the WISC-V, going beyond global index scores. This approach facilitates estimating the functional impact of EF deficits on academic performance and adaptive functioning, and it guides the planning of specific support aimed at fostering autonomy and cognitive-behavioral self-regulation [56]. Likewise, the role of the CPI as a predictor of clinical-group membership and a discriminator between presentations makes it a particularly useful indicator when behavioral symptomatology is ambiguous or when discrepancies arise between informants (family-school) [29, 57].

Nonetheless, several limitations of the study must be acknowledged. First, although the sample size was sufficient to detect medium-to-large effects, it constrains the generalizability of the findings. Additionally, the gender imbalance (overrepresentation of boys, consistent with ADHD epidemiology) limits the ability to explore potential sex-related differences. Second, comorbidity was not exhaustively controlled for (e.g., specific learning disorders, oppositional defiant disorder, conduct disorder), and such conditions may influence the cognitive profile. Finally, the cross-sectional design prevents examination of the developmental stability of the observed pattern. These considerations call for cautious interpretation of the results and highlight the need to expand the evidence in future research.

Building on these findings, several future research directions appear particularly relevant to strengthen the external validity, diagnostic sensitivity, and clinical usefulness of the proposed cognitive profile. In the first place, larger and more diverse samples are needed, including recruitment from a wider range of educational settings and geographical regions, with improved sex balance, to enhance generalizability and to enable a more fine-grained analysis of potential sex-related differences in cognitive pattern expression. In addition, incorporating more complex clinical samples, systematically characterized in terms of comorbidities, would help clarify the specificity of the profile and determine the extent to which the CPI-related pattern is uniquely associated with ADHD rather than reflecting broader neurodevelopmental vulnerability. Moreover, longitudinal approaches would be valuable to examine whether the observed cognitive configuration remains stable across developmental stages, and to test how changes in WM and PS relate to symptom trajectories, academic functioning, and adaptive outcomes from childhood into adolescence. Finally, further work could benefit from integrating advanced predictive approaches, including modern psychometrics and AI/machine-learning models, to optimize classification accuracy and to explore whether distinct cognitive subtypes can be identified within the ADHD spectrum with greater precision. Linking these cognitive markers to functional indicators, such as academic performance, socio-emotional adjustment, and response to intervention, would also allow the field to move toward more personalized psychoeducational and clinical decision-making, where cognitive profiling supports not only diagnosis but also the tailoring of support and treatment planning.

## Conclusions

The findings indicate that the WISC-V allows for the identification of a characteristic cognitive profile in ADHD, defined by lower performance on the WMI and the PSI when compared with typically developing peers. The CPI emerged as the strongest predictor for distinguishing between clinical and non-clinical groups. Accordingly, it is recommended to systematically incorporate the analysis of WISC-V cognitive indices as support for clinical judgment, without replacing the multimethod and multi-informant assessment approach required for a comprehensive evaluation of ADHD.

## Availability of Data and Materials

The datasets generated and/or analyzed during the current study are not publicly available due to privacy and ethical restrictions, but are available from the corresponding author on reasonable request and subject to approval by the corresponding Ethics Committee.
